# Quantification of metallic artifact on CT associated with titanium pedicle screws

**DOI:** 10.3389/fvets.2024.1448188

**Published:** 2024-07-31

**Authors:** M. J. Lewis, P. J. Early, R. Bergman, K. Love, N. Nelson

**Affiliations:** ^1^Department of Clinical Sciences, College of Veterinary Medicine, North Carolina State University, Raleigh, NC, United States; ^2^Synapse Veterinary Services, Fort Mill, SC, United States; ^3^K. R. Love Quantitative Consulting and Collaboration, Athens, GA, United States; ^4^Department of Molecular Biomedical Sciences, College of Veterinary Medicine, North Carolina State University, Raleigh, NC, United States

**Keywords:** vertebral column, dog, canal breach, bloom artifact, stabilization

## Abstract

**Background:**

In dogs undergoing vertebral column stabilization, post-operative computed tomography (CT) evaluates implant placement. The impact on the interpretation of metallic artifact associated with titanium implants in dogs remains to be established. Our objective was to quantify metallic artifact on CT associated with titanium pedicle screws.

**Methods:**

The study design included an *in vitro* model and a retrospective review of 11 dogs with vertebral column stabilization. Twenty four titanium pedicle screws (6 each: 2.0 mm, 2.7 mm, 3.5 mm, and 4.5 mm) were inserted into a 20% ballistic gel, and CT scan of the construct was performed. Three blinded raters used a bone window to measure the maximum width (effective size) of each screw, one rater measured effective size using an ultrawide window and 45 titanium pedicle screws (3×2.0 mm, 5×2.7 mm, 30×3.5 mm, and 7×4.5 mm) in 11 clinical cases. Effective size measurements were compared to actual screw sizes.

**Results:**

The effective size was 26.9–43.8%, 9.2–18.5%, and 21.1–30.5% larger than the actual size for the *in vitro* system (bone window), *in vitro* system (ultrawide window), and clinical cases, respectively. The mean gross difference for the *in vitro* measurements varied by implant size (*p* < 0.001) and was positively correlated with implant size (*r* = 0.846), but the mean percentage difference was negatively correlated with implant size (*p* < 0.001). Overestimation was larger for the *in vitro* model bone window compared to the ultrawide window (*p* < 0.001) and clinical cases (*p* = 0.001).

**Conclusion:**

Metallic artifact associated with titanium pedicle screws on CT resulted in an overestimation of screw size. This information might aid in the interpretation of implant placement on post-operative imaging.

## Introduction

A variety of techniques and implant constructs have been described for dogs undergoing vertebral column stabilization. Regardless of the method utilized, a key component of successful stabilization surgery is appropriate implant placement, most notably avoiding iatrogenic injury to the spinal cord or other surrounding neurovascular structures. Implantation corridors for thoracolumbar (TL) and lumbosacral (LS) vertebrae have been established in dogs using survey radiographs or computed tomography (CT) (1–5), and customized drill guides can aid in optimizing implant placement (6–15). However, in dogs undergoing TL or LS stabilization, 3.3–16.7% of titanium or stainless steel pedicle screws have been reported to penetrate the vertebral cortex medially on immediate post-operative imaging (9, 10, 15–17).

Various imaging modalities have been utilized to assess implant placement accuracy in dogs, including survey radiographs, fluoroscopy, and CT (18, 19). While CT has been demonstrated to be superior to survey radiographs in dogs and people (18, 20–23), the interpretation of acceptable placement on post-operative CT can be complicated by the metallic artifact created by the implants. The extent of the artifact varies between different types of metal, with titanium producing less artifact than stainless steel (24–26).

In people undergoing vertebral body fusion for scoliosis, titanium pedicle screws measured on CT were an average of 8–13% larger than the actual screw size. The gross amount of bloom artifact increased with increasing screw size, though it was typically <1 mm and still permitted accurate interpretation of screw placement (26). However, the implants utilized in the people were larger (ranging from 4.35 mm to 7.0 mm) than those commonly placed in dogs, and it is unclear if a similar relationship can be extrapolated to smaller implant sizes typically placed in most dogs. The metallic artifact associated with smaller diameter implants has been described in an *in vitro* CT study that reported up to a 25% overestimation of screw size using a standard CT protocol (27). A mix of stainless steel and titanium condylar screws were utilized (including only 1 each of 2.4 mm, 2.7 mm, 3.5 mm, and 4.5 mm titanium locking or transcondylar screws), and the reliability of measurement data was not reported (27). Therefore, as titanium pedicle screws are increasingly being utilized for vertebral column stabilization in dogs, more details are needed regarding the metallic artifact visualized on CT to facilitate accurate interpretation of post-operative imaging.

The objectives of this study were to quantify the metallic artifact on CT associated with titanium screws of a pedicle screw-rod fixation (PSF) system designed for dogs using an *in vitro* model and retrospectively from clinical cases in which the vertebral column was stabilized. We hypothesized that the *effective* size (implant + artifact) would be larger than the *actual* implant size in similar proportions to that reported for larger screws placed in people.

## Materials and methods

The study design included an *in vitro* model and a retrospective review of clinical cases from 2022 to 2023 in which the PSF system was utilized to stabilize the vertebral column.

### *In vitro* model construction

**Figure 1:**
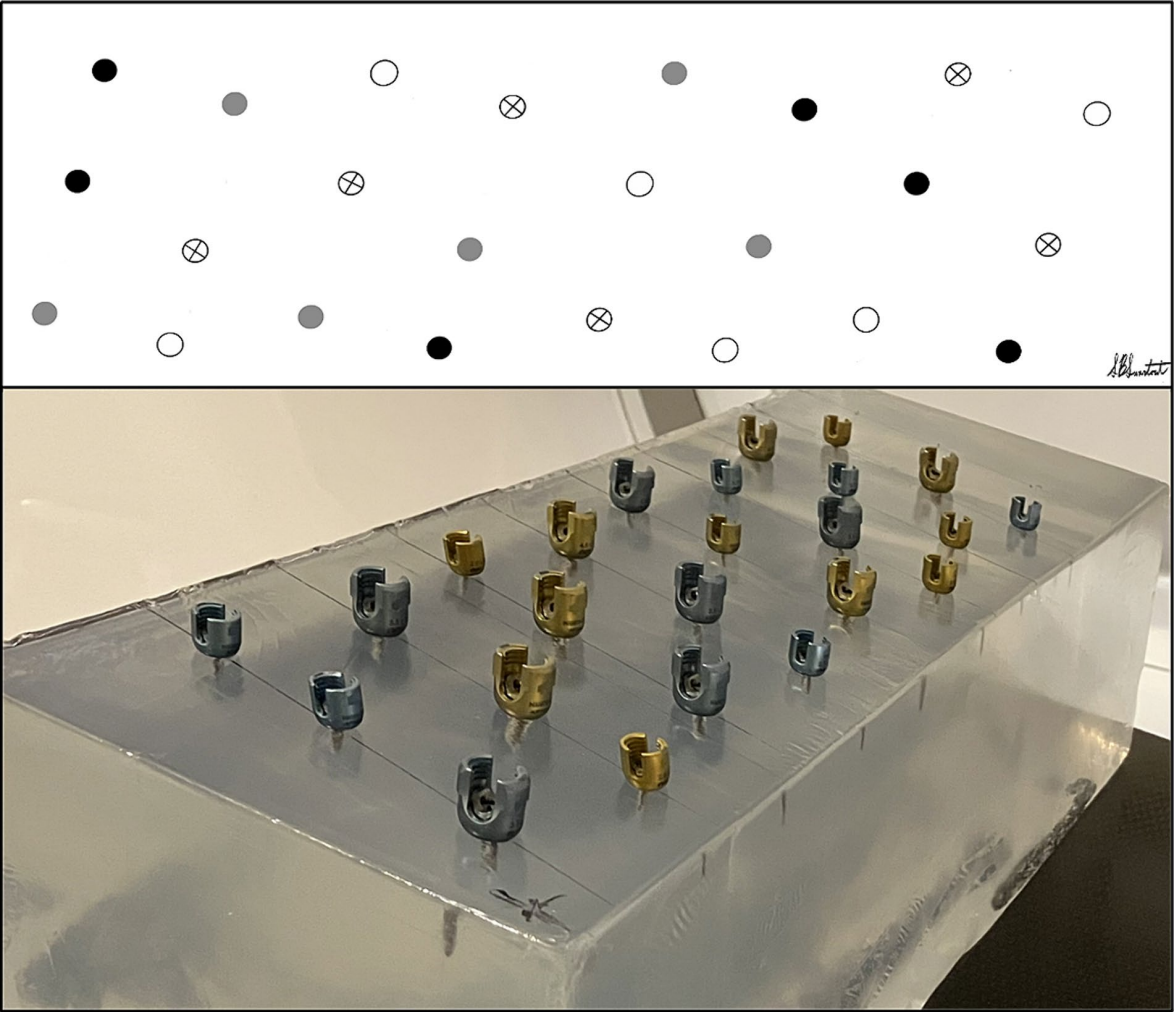
Arrangement of pedicle screw placement into a 20% ballistic gel block.

The *in vitro* construct consisted of a 20% ballistic gel block of 20″ x 6″ x 6″ dimensions (Clear Ballistics, Greenville, SC). Twenty four titanium Dual Lead Pedicle Screws (OrthoMed North America, Inc., Redwood City, CA) ranging in size from 2.0 mm to 4.5 mm were placed into the ballistic gel. Specifically, there were 6 each 2.0 mm x 18 mm, 2.7 mm x 18 mm, 3.5 mm x 25 mm, and 4.5 mm x 25 mm. Manufacturer specifications report a screw size precision of +/− 0.013 mm. All screws were placed by one investigator (PJE) who was not involved in measurements, and the order of placement was randomized using a random number generator (chat.openai.com). To ensure even spacing during placement and to avoid overlapping streak artifacts between adjacent screws, a grid was created on the surface of the gel using a goniometer and ruler. It consisted of eight rows of three screws each, staggered along a diagonal for a given row, and a permanent marker was used to mark each screw location (Figure 1). The gel surface was punctured with a 1.00 mm K wire. Using a Hall cordless drill, holes were then drilled for each screw as follows: 1.5 mm drill bit for 2.0 mm screws, 2.0 mm drill bit for 2.7 screws, 2.5 mm drill bit for 3.5 mm screws, and 3.2 mm drill bit for 4.5 mm screws. Each screw was then placed perpendicular to the surface of the gel, with only the tulip remaining above the surface.

### *In vitro* model image acquisition and measurements

**Figure 2:**
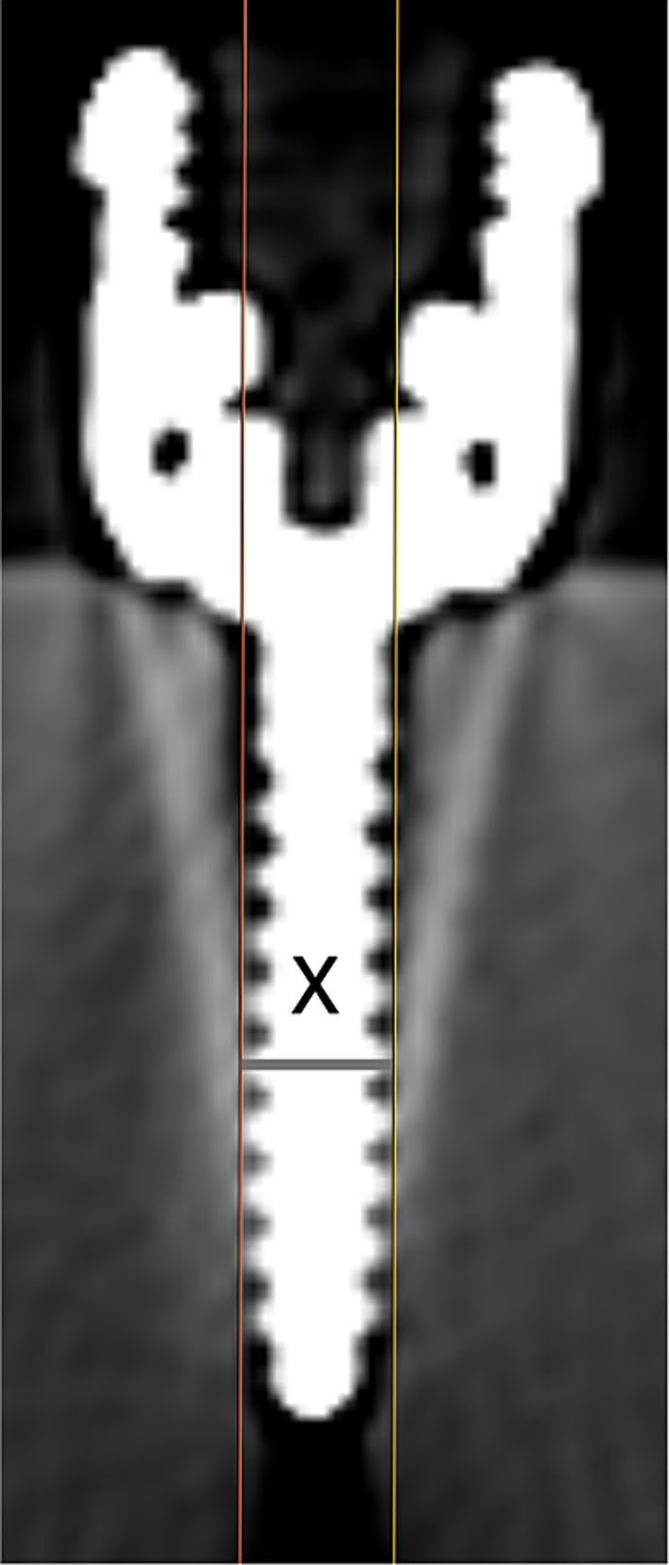
Technique to measure screw width, where ‘x’ designates the maximum width for this screw on this slice.

Following screw placement, a non-contrast CT scan of the construct was performed (Siemens Somatom Perspective 64, Forchheim, DE), with 1 mm slice thickness, 130 kVp, 132 mA, and a field of view large enough to encompass the ballistic gel block. Metallic artifact reduction software was not utilized. Three investigators (MJL, NN, and RB) blinded to screw size and placement order independently measured each of the 24 screws. Using the technique described by Elliott et al. in 2014 (26), each screw was measured in the transverse plane using non-contrast-enhanced images of 1 mm slice thickness in a bone window [window width (W) 2000, window level (L) 500] and with a zoom factor of 7x. For each slice in which a given screw appeared, two parallel lines were drawn along the outer edge of the threads on each side of the implant shaft. A perpendicular line connecting the parallel lines was created to measure the implant width for that slice (Figure 2). The ‘effective size’ for each screw was defined as the largest width measurement across slices and represented the implant plus the associated artifact. Measurements and determination of ‘effective size’ were repeated for all screws in the gel. The manual manipulation of window level or width was not permitted. Two weeks after the first measurements were performed, one investigator (MJL) repeated the measurements on a randomly selected subset of eight (33%) screws. For comparison to the typical bone window, one investigator (MJL) also repeated the measurements on all screws using an ‘ultrawide’ window (W 20,000, L 3000).

### Clinical cases with PSF implants

The medical record system at the NC State University Veterinary Hospital was searched to identify dogs in which the PSF titanium screws were utilized to stabilize the vertebral column between T1 and S3 and where an immediate post-operative CT scan was performed. Cases were excluded if the size of the implants that were placed could not be determined from the medical record.

One investigator (PJE) reviewed the medical record for each dog and recorded the location of stabilization and the size and location of screws that were placed. A second investigator (MJL), without prior knowledge of screw sizes, reviewed each post-operative CT scan. Screw measurements were performed as outlined for the *in vitro* system in the transverse plane, using non-contrast-enhanced images of 1 mm slice thickness, a bone window (W 2000, L 500), and a zoom factor of 7x. The ‘effective size’ for each implant was similarly defined as the largest measured width of the shaft of that implant. The placement of each implant was subjectively assessed using the bone window and the modified Zdichavsky classification system (3, 9, 28), focusing on vertebral canal penetration. Grade I was defined as the screw being fully contained with the bone on all slices with no communication with the vertebral canal. Grade IIa was defined as the screw being in contact with or having partial penetration of the medial vertebral cortex on at least one slice. Grade IIb was defined as an overt violation of the vertebral cortex and the screw being within the vertebral canal on any slice (9).

### Statistical analysis

Analyses were performed using SPSS (SPSS Statistics v29, IBM, Chicago, IL). Continuous data were presented as mean and standard deviation after confirmation of normality using a Shapiro–Wilk test. Implant placement (Grade I, IIa, or IIb) was tabulated for the clinical cases, and data were presented descriptively. For each screw size, the mean (SD) individual and mean (SD) group ‘effective size’ (implant + artifact) values were calculated from the CT measurements obtained using the bone window. Using the manufacturer’s reported screw size and the group measurement data, the gross difference and the ratio (i.e., percentage change) between the ‘effective size’ and ‘actual size’ were determined for the four screw sizes. Since gross differences fit the statistical model better than ratios, gross differences were utilized for subsequent analyses. Raters were compared to each other (inter-rater), and repeat measurements from the same rater (intra-rater) were compared using a two-way analysis of variance (ANOVA), including screw size as a covariate. Furthermore, the variability in measurements between raters (inter-rater) and within one rater (intra-rater) was compared using Levene’s test. Since there was no measurable correlation among gross artifact measures of the same screws or raters, a mixed effect model was eliminated in favor of simple models incorporating fixed effects only. A one-way ANOVA was used to compare the gross difference by screw size. *Post-hoc* pairwise comparisons were then used to compare mean gross differences for pairs of screw sizes (all combinations), and Pearson’s correlation was used to evaluate the relationship between the gross artifact size and implant size.

The mean (SD) of ‘effective size’ and the gross difference and ratio (i.e., percentage change) between the ‘effective size’ and ‘actual size’ were also calculated for the measurements obtained using the ultrawide window and the clinical cases. A two-way ANOVA was used to compare the gross difference for the bone versus ultrawide windows and as measured for the *in vitro* system (bone window) versus the clinical cases. For all analyses, a *p*-value of <0.05 was considered significant, with multiple comparisons corrected using the Bonferroni method where indicated.

## Results

### *In vitro* model

Individual and group effective size measurements are displayed in Table 1. Comparing the raters, there was no significant difference in the mean group differences by rater, after adjusting for the effects of screw size (*p* = 0.383). Additionally, there was no evidence that any raters had different variability in their measurements compared to the other raters (*p* = 0.353). For the intra-rater comparison, the mean gross difference (averaged across all screw sizes) was smaller for the repeated measurements (0.889 mm, SE 0.043) compared to the initial measurements (1.011 mm, SE 0.043), but this difference was not significant (*p* = 0.059). The variability around that rater’s average for each screw size was also smaller for the repeated measurements compared to initial measurements, with borderline significance (*p* = 0.05). Across the four screw sizes, the effective size was 26.9 to 43.8% larger than the actual size. As implant size increased, the mean gross size of the artifact averaged across the raters (i.e., the gross difference between effective and actual size) generally increased, and the mean gross difference was positively correlated with implant size (*r* = 0.846, *p* < 0.001). For comparisons between all pairs of screw sizes except for 2.7 mm to 2.0 mm screws (*p* > 0.05), the larger screw size had a significantly larger gross difference compared to the smaller screw (*p* ≤ 0.048, all comparisons). However, the ratio of effective size to actual size (i.e., the percentage overestimation of size) decreased as screw size increased. The mean ratio was negatively correlated with implant size (*r* = −0.794, *p* < 0.001).

**Table 1:** Individual and group ‘effective size’ measurements (in mm) were calculated using the bone window and presented as mean (SD) for each screw size.

	Screw size			
Rater	2.0 mm (*N* = 6)	2.7 mm (*N* = 6)	3.5 mm (*N* = 6)	4.5 mm (*N* = 6)
Rater #1	2.87 (0.16)	3.52 (0.09)	4.65 (0.09)	5.70 (0.12)
Rater #2	2.77 (0.21)	3.55 (0.08)	4.64 (0.12)	5.84 (0.10)
Rater #3	2.99 (0.14)	3.55 (0.11)	4.45 (0.11)	5.59 (0.15)
Group	2.88 (0.18)	3.55 (0.08)	4.58 (0.14)	5.71 (0.16)
Group gross (%) size difference	0.88 (43.8%)	0.85 (31.4%)	1.08 (30.8%)	1.21 (26.9%)
Group Minimum	2.53	3.43	4.32	5.41
Group Maximum	3.23	3.68	4.87	5.96

**Figure 3:**
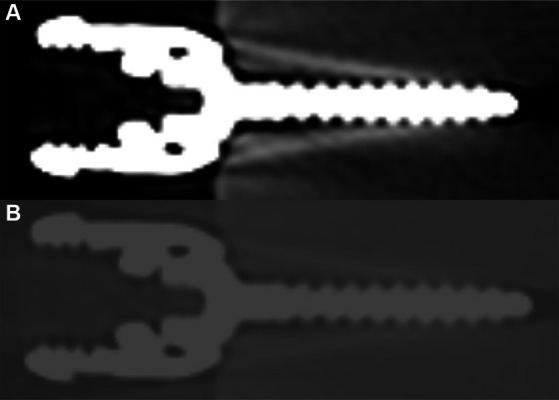
Representative example of a 3.5 mm titanium pedicle screw utilizing **(A)** bone window (W 2000, L 500) and **(B)** ultrawide window (W 20,000, L 3000).

Effective size measurements obtained using the ultrawide window are outlined in Table 2, and a representative example of the same screw utilizing a bone window versus an ultrawide window is depicted in Figure 3. Across the four screw sizes using the ultrawide window, the effective size was 9.2 to 18.5% larger than the actual size. Comparing the two window types, the percentage difference in artifact size was 11.3 to 25.3% larger for the bone window, with a mean gross difference in artifact size of 0.552 mm across implant sizes. The mean gross difference between effective and actual size was significantly larger for measurements performed using the bone window compared to the ultrawide window (*p* < 0.001), with no evidence that the difference in artifact size between the two windows depended on the size of the implant (*p* = 0.234).

**Table 2:** ‘Effective size’ measurements (in mm) were calculated by one investigator on a repeat subset of the screws using the bone window and, on all screws, using the ultrawide window for the ballistic gel construct, and using the bone window for clinical cases.

Measurement type	Screw size			
	2.0 mm (*N* = 2)	2.7 mm (*N* = 1)	3.5 mm (*N* = 3)	4.5 mm (*N* = 2)
Repeat bone window	2.71 (0.11)	3.48 (NA)	4.52 (0)	5.58 (0.05)
Gross (%) size difference	0.71 (26.2%)	0.78 (22.4%)	1.02 (29.1%)	1.08 (24.0%)
	2.0 mm (*N* = 6)	2.7 mm (*N* = 6)	3.5 mm (*N* = 6)	4.5 mm (*N* = 6)
Ultrawide Window	2.37 (0.08)	2.95 (0.05)	4.01 (0.07)	5.20 (0.06)
Gross (%) size difference	0.37 (18.5%)	0.25 (9.2%)	0.51 (14.4%)	0.70 (15.6%)
	2.0 mm (*N* = 3)	2.7 mm (*N* = 5)	3.5 mm (*N* = 30)	4.5 mm (*N* = 7)
Clinical cases (Bone window)	2.60 (0.06)	3.45 (0.19)	4.37 (0.23)	5.45 (0.35)
Gross (%) size difference	0.61 (30.5%)	0.75 (27.9%)	0.87 (24.8%)	0.95 (21.1%)

Values are presented as mean (SD) for each screw size.

### Clinical cases

Eleven dogs were identified that met the inclusion criteria. The median age was 2 years (range: 6 months to 9 years), and the mean body weight was 25.4 kg (+/− 14.8 kg). The reason for vertebral column stabilization was traumatic fracture/subluxation in eight dogs, degenerative LS stenosis in one dog, and bilateral decompressive hemilaminectomy in two dogs. A total of 45 titanium pedicle screws were evaluated, ranging in size from 2.0 mm to 4.5 mm, with 30 out of 45 (67%) screws being 3.5 mm in diameter. Three to six implants were placed in each dog, typically with one to two placed per vertebrae. The location of stabilization ranged from T9-S1, including eight dogs stabilized between T9 and L4 and three dogs stabilized at L7-S1. Implant placement was classified as Grade I for 36 implants, Grade IIa for 9 implants in 7 dogs, and Grade IIb for 0 implants. For all nine implants designated as Grade IIa, the screw was characterized as being in contact with the medial vertebral cortex with no or minimal canal penetration. Grade IIa placement was noted for at least one implant of each size and for both TL and LS stabilizations. No implants were surgically revised except for two screws in S1 noted in proximity to the L7-S1 intervertebral foramina in a single dog. No adverse effects attributed to implant placement were noted for any dog.

**Figure 4:**
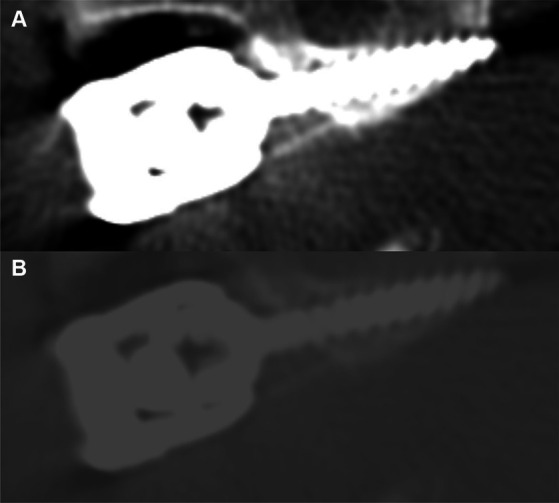
Representative example of a 3.5 mm screw from a clinical case utilizing **(A)** bone window (W 2000, L 500) and **(B)** ultrawide window (W 20,000, L 3000).

Effective size measurements are outlined in Table 2. Across the four screw sizes, the effective size was 21.1 to 30.5% larger than the actual size, and the mean gross size of the artifact increased as the screw size increased. Comparing the *in vitro* system (using the bone window) to the clinical cases across implant sizes, the mean gross difference in artifact size was 0.213 mm. The difference between the effective and actual size of the screws was significantly larger when measured using the *in vitro* system versus in the clinical cases (*p* = 0.001), with no evidence that this is dependent on the size of the implant (*p* = 0.577). A representative example of a screw from a clinical case is depicted in Figure 4, showing the difference in metallic artifact and visualization of anatomic details between bone and ultrawide windows.

## Discussion

The metallic artifact associated with titanium pedicle screws on CT produced an effective size that was larger than the actual screw size for all screws measured. Since this overestimation was significantly larger for the *in vitro* construct compared to the clinical cases, it is likely that the artifact associated with implants placed in the vertebrae of clinical cases will be less pronounced. These results will aid veterinary neurosurgeons in interpreting implant placement on post-operative CT, perhaps especially for implants where possible canal violation or foraminal impingement is within the degree of overestimation, as demonstrated in this study.

The ballistic gel construct provided a simple model system to quantify screw size and address the question of implant-associated artifact. Measurements were comparable across raters with regard to the gross amount of measured artifact and the variability in their measurements, although no inexperienced clinicians were involved with this study. Prior imaging-based morphometric studies have yielded variable results regarding the effect of experience (4, 29–32). However, the measurement technique used in this study was straightforward and did not require particular knowledge of CT or implants. Coupled with the lack of evidence that raters differ significantly from each other, this suggests that the measurement data would likely remain representative across raters with a range of clinical experience.

While the *in vitro* system was a feasible, clinically applicable model, measurements on screws in the ballistic gel were consistently larger for comparable screw sizes in the clinical cases. The reason for the effective size measurements being smaller for the clinical cases compared to the ballistic gel is not clear and is likely multifactorial. One consideration is that the measurements for the clinical cases were performed after all the ballistic gel measurements. There could be a learning curve with the *in vitro* measurements akin to ‘training,’ leading to more accurate subsequent measurements, as has been suggested in another morphometric-based imaging study (29). While not significant, the fact that repeated measurements from the same rater were, on average, smaller than the original measurements could lend additional support for this assertion. Another possibility is that effective size measurements were impacted by the difference in the interface between the implant and the surrounding medium, soft tissue attenuating for the gel compared to mineral attenuating for the clinical patients. It has previously been noted that the bone of the tympanic bulla in dogs appears thicker on CT when fluid versus air-filled, which is suggested to be due, in part, to the fluid versus air interface with the bone (33). However, those findings would suggest that measurements in the clinical patients should have had a greater overestimation of artifact. Regardless, there could be other ways in which the relationship between the implant and the adjacent medium influenced the measurements.

The effective screw size was larger than the actual screw size for all measured screws, partially confirming our hypothesis. However, the percentage overestimation of screw size for both *in vitro* (26.9–43.8%) and clinical cases (21.1–30.5%) was larger than the 8–13% reported for titanium pedicle screws placed in people (26). Overestimation was comparable or larger compared to the ≤25% reported from *in vitro* measurements of a mix of titanium and stainless steel condylar screws of sizes typically used in dogs, but direct comparison is challenging since that study only evaluated four titanium condylar screws, and metallic artifact was not the primary focus (27). Despite the reasonably large percentage difference, the gross amount of bloom artifact was small (<1.2 mm) and generally increased with increasing implant size. This is in line with measurements reported in people, but that study also reported that scatter increased as implant size got larger (26), whereas we identified that the percentage difference between actual and effective size generally decreased as screw size got larger. These discrepancies might relate to differences in methodology and an outsized impact of magnification on the spatial resolution of smaller implants. Our results suggest that the metallic artifact might be overestimated to a greater degree for small implants, which has clinical implications since they are utilized in patients with the smallest implant corridors.

We also investigated an ultrawide window (W 20,000, L 3000), which significantly reduced the metallic bloom artifact. This finding was similar to prior experimental studies of metal implants, where extended-scale CT protocols using a markedly widened window (W up to 50,000) significantly reduced artifacts (27, 34). While the effective size measurements using the ultrawide window were much closer to the actual screw sizes, there were drawbacks to this approach. The edges of the screw could be identified clearly on the ultrawide window, but there was a reduction in contrast of the rest of the image. This limited the ability to discern adjacent bone anatomy and mitigated much of the benefit of the reduced artifact. This is consistent with an experimental study using porcine tibias in which some artificially created bony lesions (e.g., to mimic osteolysis due to implant-associated infection) were not identified on the extended scale CT that were visible using a standard window (34). Metallic artifact reduction algorithms have also been developed but with variable success at decreasing distortion (35–39). Some protocols can also result in an underestimation of implant size and reduce contrast and the diagnostic quality of the rest of the image (37). Currently, such methods are not widely available, and their clinical applications have not been established for surgical implants placed in veterinary patients. However, specialized CT protocols involving adapted windowing, metallic artifact reduction algorithms, and other techniques are worthy of further exploration to optimize the imaging interpretation of vertebral implants.

While this study was not primarily designed to assess canal breaches or their clinical consequences, the results have implications for assessing canal violations. Based on the measurement data, it is possible that some borderline (Grade IIa) violations, as judged on CT, might not be penetrating the medial vertebral cortex. Similarly, overt canal violation by a screw would also likely be somewhat overestimated on CT, perhaps particularly for the smallest implants. Additionally, no implants that were judged to be Grade IIa had any clinical consequences identified, and none were revised due to canal violation. This is in keeping with prior reports of vertebral column stabilization in dogs where pedicle screws with suboptimal placement (but that were not revised) demonstrated no clinically detectable detrimental effects (15–17). Our findings provide tangential support for the assertion that borderline or mild apparent canal violation on CT is likely acceptable in dogs.

Limitations of this study include the *in vitro* nature of the majority of the measurements and the relatively small number of screws assessed in dogs, especially for sizes other than 3.5 mm diameter. The data should be interpreted cautiously when extrapolating more broadly to clinical cases, but it can be anticipated that the artifact *in vivo* will likely be smaller than our *in vitro* measurements for the same-sized implant. Additionally, this study only assessed a single type of titanium pedicle screw. As such, these results might not be representative of other titanium implants and do not apply to stainless steel screws that are currently more widely utilized for vertebral column stabilization in dogs.

In conclusion, the metallic artifact on CT produced by titanium pedicle screws resulted in an overestimation of the actual screw size. While the artifact was grossly small, it was relatively more pronounced for the smaller-sized implants. These quantitative findings will aid in interpreting CT scans following vertebral column stabilization in dogs.

## Data availability statement

The raw data supporting the conclusions of this article will be made available by the authors, without undue reservation.

## Ethics statement

Ethical approval was not required for the studies involving animals in accordance with the local legislation and institutional requirements because the work was performed *in vitro* or was retrospective in nature using medical record data only. Written informed consent was not obtained from the owners for the participation of their animals in this study because only Retrospective data obtained from medical records was used.

## Author contributions

ML: Conceptualization, Data curation, Formal analysis, Methodology, Writing – original draft, Writing – review & editing. PE: Conceptualization, Data curation, Formal analysis, Methodology, Writing – original draft, Writing – review & editing. RB: Data curation, Formal analysis, Writing – review & editing. KL: Formal analysis, Writing – review & editing. NN: Data curation, Formal analysis, Methodology, Writing – review & editing.
